# The Hi-Audio online platform for recording and distributing multi-track music datasets

**DOI:** 10.1186/s13636-026-00459-0

**Published:** 2026-03-31

**Authors:** José M. Gil Panal, Aurélien David, Gaël Richard

**Affiliations:** LTCI, Télécom Paris, Institut Polytechnique de Paris, Paris, France

**Keywords:** DAW, MIR, Multitrack, Music, Audio, Latency, Browser, Recording, Dataset, Usability

## Abstract

This paper introduces the Hi-Audio online platform, an open-source tool designed to support musicians and researchers in the field of Music Information Retrieval (MIR). The platform enables the recording, uploading, and sharing of multitrack musical compositions, aiming to build an open-access audio database to advance research in music technology. Uploaded audio files are automatically analyzed upon synchronization with the server, leveraging signal processing techniques and machine learning models to generate rich metadata. The platform facilitates remote and asynchronous collaboration via a web-based interface accessible at hiaudio.fr. Furthermore, a novel built-in method for accurate and robust round-trip latency estimation in the browser is proposed and integrated into the platform, demonstrating its applicability in real-world distributed recording scenarios. Finally, an initial user evaluation with musicians was conducted to assess usability and practical relevance under realistic usage conditions. The evaluation combined task-based performance analysis with standardized usability and workload measures. The results indicate high task completion rates for core recording functions and show that the platform can be used effectively by musicians with minimal prior training.

## Introduction

The approach to music production and its creative process has dramatically evolved over recent decades, driven by technological advancements in the digital era and the widespread adoption of computers and affordable electronic devices. As a result, software and tools have become more accessible, frequently offered as ready-to-use solutions that demand minimal technical expertise and limited resources.

Recent trends towards the development of artificial intelligence (AI)-based tools for specific music-related applications have opened new avenues for innovative composition methods [1]. Despite these advancements, creating such AI models and technologies remains resource-intensive, not only due to high computational demands (e.g., memory, processing capabilities) for training purposes but also because of the substantial volume of domain-specific data required.

Compared to other domains, such as image processing, the MIR field continues to experience a shortage of suitable datasets, particularly annotated multitrack data [2–4]. Data is essential for training, creating, and adapting AI algorithms and models for tasks such as source separation or music generation [5].

Intellectual property and copyright laws play a significant role in shaping how musical works are distributed and accessed online. These legal frameworks often hinder the open sharing of resources, thereby encouraging the adoption of alternative licensing models—such as CC (Creative Commons)—to foster collaboration and reuse of content [6]. Nevertheless, the availability of musical datasets remains limited in both scope and diversity. Many existing collections^1^ tend to focus on specific genres, predominantly Western music^2^, which constrains the development of culturally inclusive AI models. This limitation is further compounded by the nature of certain music traditions, such as classical or folk genres, where performances are typically recorded live with multiple instruments played simultaneously, making the production of multitrack recordings more challenging.

At the same time, technological progress—particularly in web technologies—offers promising alternatives. Modern web browsers are now equipped with advanced and versatile audio capabilities, enabling cross-device audio capture and supporting the creation of browser-based Digital Audio Workstations (DAWs) [7]. Despite these innovations, many existing platforms and tools for music recording, distribution, and collaboration remain proprietary or commercial in nature, restricting public access to both their source code and the content they generate.

This article presents the Hi-Audio online platform and addresses the challenges involved in developing an open-source system for massive audio collection, aimed at making musical databases openly available to the general public and particularly to the scientific community. Ethical considerations are emphasized, not only to prevent bias in the generated data but also to protect intellectual property, user identity, and privacy. Additionally, mechanisms are provided to facilitate reuse, cooperation, and data exploitation in line with FAIR principles of science^3^.

Leveraging web-based technologies, the platform design aims to lower entry barriers for musicians with diverse backgrounds by a minimalist and simplistic interface. It encourages participation regardless of users’ prior experience, focusing instead on their interpretative and creative capabilities.

**Fig. 1:**
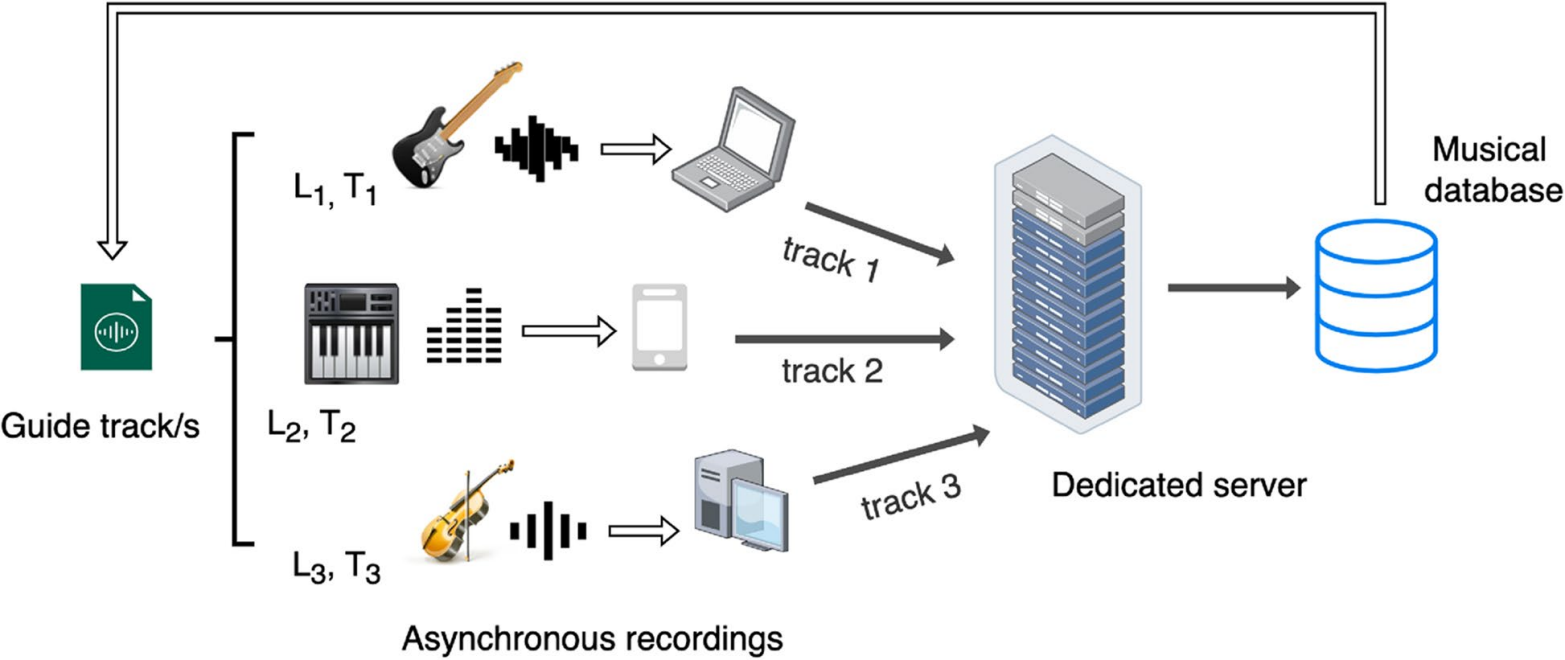
Illustration showing the overall recording scheme of Hi-Audio online platform. The recording process is distributed, taking place at different locations (L1 $\ne$ L2 $\ne$ L3) and at different time points (T1 < T2 < T3). All data is stored and annotated on a dedicated server. A guide track hosted in the database, may serve as a reference to enable synchronized performance by each participant

This paper details the implementation, features, and necessary infrastructure of the Hi-Audio system (illustrated in Fig. 1), describing various techniques employed, including (1) latency correction during audio input, (2) automatic labeling and annotation of audio tracks with relevant metadata, and (3) server-side audio file compatibility and compression to optimize data retrieval and download speeds, typically hindered by heavy formats like WAV.

While the primary focus of this work is on the design and implementation of a collaborative audio recording platform, understanding how musicians interact with such a system in practice is essential for validating its applicability. For this reason, apart from the public demonstrations [8, 9] and participatory events^4^, the present work also includes a preliminary user evaluation study, aimed at assessing first-time usability, task performance, and perceived workload under realistic usage conditions.

The main contributions of the presented work, discussed in subsequent sections, include:

1. Providing an open ecosystem that facilitates the asynchronous creation of annotated, open musical databases with isolated audio tracks covering a broad spectrum of instruments and genres through a unified interface. It thereby promotes scientists and researchers to collaborate and address the scarcity of datasets in the MIR domain.
2. Ensuring content accessibility and distribution through open licenses, user roles and privacy controls, thus encouraging user participation, interaction, and collaboration in the construction of a comprehensive music catalog.
3. Proposing and integrating a novel browser-based round-trip latency estimation and compensation method based on maximum length sequence (MLS) signals, validated across devices, operating systems, and browsers, and implemented within the Hi-Audio open-source web DAW.
4. Presenting an initial user study that evaluates the platform from the perspective of end users, providing preliminary insights into usability, practical adoption, and interaction with the system in realistic usage scenarios.

## Related works

This section discusses existing systems and domains related to Hi-Audio’s functionalities and objectives, particularly focusing on internet-based music collaboration, and justifies the necessity for developing a tailored solution to construct suitable datasets for MIR applications; note that while these goals partially overlap with the scope of platforms such as Freesound [10, 11], Hi-Audio specifically targets the structured creation and annotation of multitrack music data collections.

### Motivation and positioning

It is important to clarify that the motivation behind Hi-Audio is to establish an open ecosystem for the collection and distribution of multitrack compositions, distinctly unrelated to digital platforms focused purely on audio streaming, such as Spotify, Soundcloud, or Bandcamp. Furthermore, Hi-Audio is not designed for real-time music execution like Audiomovers ListenTo^5^, JamKazam^6^, Ninjam by Cockos^7^(creators of Reaper), or JamNSync [12]. Instead, Hi-Audio is a research-oriented distributed musical creation tool characterized by the voluntary, asynchronous, and remote participation of musicians in content generation. Consequently, functionalities promoting user interaction within a single composition (authentication, invitations, roles, permissions) and features supporting catalog creation and organization (collections, annotations) have been emphasized in the system’s design and conception. Additionally, privacy levels (public and private) and flexible licensing options—such as CC BY 4.0^8^—have been essential considerations to ensure proper authorship attribution and to facilitate content reuse, given its online publication and distribution model.

The proposed platform relates also to the work present in the emerging field of the Internet of Musical Things (IoMusT), which studies networked musical devices, web technologies, and cloud infrastructures supporting music creation, learning, and collaboration. Prior work in IoMusT has explored instrument–cloud interactions [13], distributed DSP services [14], ethical implications of connected musical ecosystems [15], and collaborative web-based music systems for education and performance [16, 17]. While much of this research focuses on real-time interaction, smart instruments, or synchronous performance, Hi-Audio addresses complementary IoMusT challenges such as scalable web-based audio capture, metadata annotation and reproducibility. By supporting asynchronous collaboration for rehearsal, learning, and dataset creation within the browser, the platform situates itself at the intersection of IoMusT, Web Audio technologies, and ubiquitous music practices.

A research priority in Hi-Audio is the accurate, natural capture of musical instrument sounds, typically using microphones (external or integrated), guiding the development approach and prioritizing this objective over advanced user-level features commonly found in other platforms, such as sound effects, MIDI support, plugins, filters, or audio sample libraries. Thus, Hi-Audio is intended for distributing completed tracks/stems and acquiring audio sources before processing rather than comprehensive audio editing or final production.

### Online DAWs

DAWs, particularly commercial ones, are extensively used both professionally and domestically due to their ability of recording audio using modern electronic devices and media. Between 2013 and 2015, the first online DAWs appeared using web standards such as HTML5 and JavaScript, with Soundtrap [18, 19] (2013) as one of the pioneers. Subsequently, many commercial platforms emerged, notably Audiotool [20] (2008, initially based on obsolete Flash technology), Soundation [21], Amped Studio [22], and BandLab [23] (2015). Additionally, various platforms have promoted musician collaboration online, including Blend.io (2013, discontinued in 2024), Splice (2013, studio collaboration tool discontinued in June 2023). In both cases, collaboration was facilitated by synchronizing local files stored on desktop workstations such as Ableton or GarageBand. In this context, proprietary solutions like Avid Cloud^9^, Muse^10^, and Satellite by Mixed in Key^11^ have taken the lead. Kompoz^12^ (2007), a web application promoting online music collaboration, lacks a multitrack viewer for recording; collaboration is instead based on a social network model for uploading and downloading files, although it includes an audio player for listening to existing mixes.

The comparative Table 1 highlights the various cloud-based workstation services mentioned, analyzing features targeted by Hi-Audio. In web applications similar to Hi-Audio, for example Soundtrap, Amped Studio, or BandLab, collaboration does not follow a role-based or permission-level structure. However, varying levels of collaboration or access control are available in comparable platforms such as AudioTool and Soundation. When it comes to open-source alternatives, the number of available options is significantly limited, with only a few notable services, including GridSound [24] (2015), Wavacity [25] (2022), and WAM-Online Studio [26] (2023), none of which currently support online collaboration. In GridSound, users can clone projects created by others but cannot work jointly on the same, and recording audio input (e.g., from a microphone) is not supported. Wavacity is a web-based port of Audacity made possible through WebAssembly [27]. It closely replicates the desktop version of Audacity but lacks connection to any data server, meaning users cannot save their work. WAM-Online Studio, being a more recent and experimental utility, currently does not support user registration or account creation.

**Table 1:** The comparative table shows various cloud-based online platforms for music creation built using DAWs, alongside the features implemented in Hi-Audio designed to foster user collaboration and content dissemination. Columns from left to right represent: 1) availability as open-source software, 2) capability for creating collections or organizing compositions by criteria, 3) inclusion of annotations or metadata at track level, 4) privacy or visualization levels (e.g., public access), 5) collaboration among users on the same composition, 6) definition of collaborator roles (administrator, guest, etc.), 7) ability to duplicate an existing composition for use as a template, 8) whether music is available under CC or not

App	Open-source	Collections	Custom metadata	Privacy levels	Collaboration	User roles	Clone	Music CC
Soundtrap [28]	No	Yes^a^	No	No	Yes	No	Yes^b^	No
Amped Studio [22]	No	No	Yes^c, d^	Yes^d, e^	Yes^d^	No	Yes^d^	No
Bandlab [23]	No	No	No	Yes^e^	Yes	No	Yes	No
Soundation [21]	No	No	No	Yes^e^	Yes	Yes	Yes	No
Audiotool [20]	No	No	Yes^c^	Yes	Yes	Yes	Yes	Yes
GridSound [24]	Yes	No	No	No	No	No	Yes	Yes
WAM-Online Studio [29]	Yes	No	No	No	No	No	No	No
Wavacity [25]	Yes	No	No	No	No	No	No	No
Hi-Audio	Yes	Yes	Yes	Yes	Yes	Yes	Yes^f^	Yes

^a^Soundtrap does not allow creating collections (referred to as folders) within other collections. Additionally, neither the collections nor the compositions can be publicly explored

^b^Soundtrap only allows cloning default templates, known as demo projects, that they provide

^c^It allows adding custom tags or keywords at the composition (or song) level, but not for individual tracks within a composition

^d^In Amped Studio, a song can be set to public or private, but a premium account is required. The same applies to collaboration, sharing, and cloning

^e^The published content is accessible exclusively to registered users and is not publicly available

^f^In Hi-Audio, templates can be cloned by any user; however, currently only DB managers can mark compositions as templates, see Sects. 3 and 3.4

In addition to the platforms listed above, a newly released open-source web-based DAW, openDAW^13^, deserves mention. OpenDAW does not rely on a backend service; consequently, it does not provide user registration, and project files must be stored locally or synchronized via third-party services like Google Drive or Dropbox. Importantly, openDAW adopts DAWproject^14^, an open and standardized exchange format designed to facilitate interoperability between DAWs, reflecting a growing interest in data portability within web-based audio production tools.

Regarding the integration of AI into workflows, several online services exist, such as the commercial platforms Moises.ai^15^ and Amped Studio, or academic initiatives like Songle [30] by AIST (Japan). These services integrate analysis using machine learning (ML) models for tasks ranging from source separation, tempo estimation, or extraction of other relevant musical information including chords or melody. In Hi-Audio, as previously stated, the sole purpose for employing ML models is to facilitate the annotation of audio tracks, a typically labor-intensive task crucial for supervised model training. Further implementation details are provided in Sect. 4.2.1.

## Main features

**Fig. 2:**
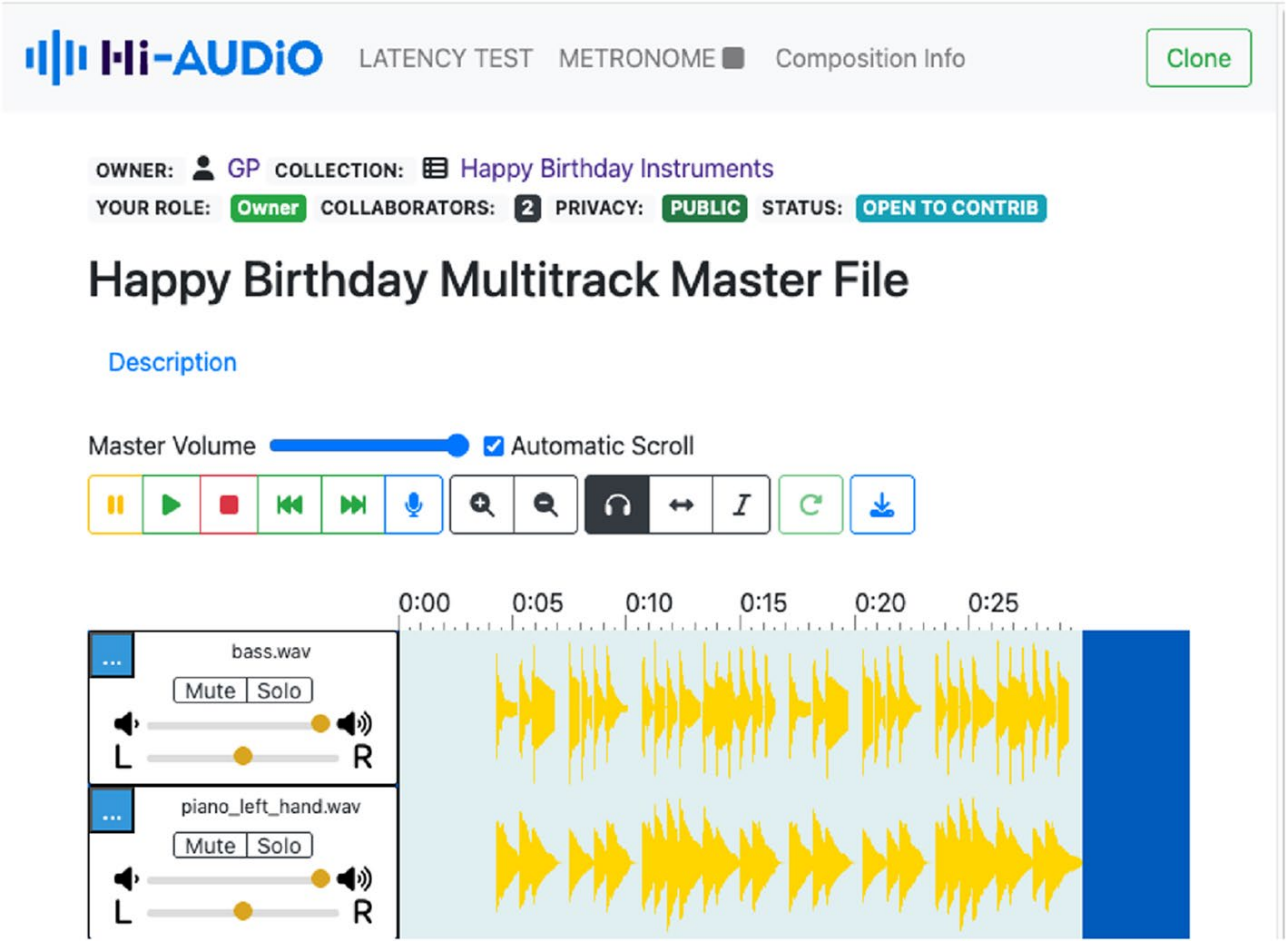
The Hi-Audio platform UI multitrack editor based on waveform-playlist. The top navigation bar displays the main options available on the Composition page: latency test, metronome, composition information and optionally a Clone button that allows users to duplicate compositions designated as templates in the database

This section presents the main features of the Hi-Audio platform that distinguish it from related initiatives discussed in Sect. 2. It provides an overview of the core user-facing functionalities and high-level design choices supporting content creation, independent of implementation details. The web application is organized into several primary views that structure user interaction and navigation, including the Home, Profile, and Composition pages, each supporting specific actions related to content browsing, account management, and multitrack audio creation. One of the key and distinctive features of the Hi-Audio online utility is its method for estimating round-trip latency. This method enables accurate temporal alignment of audio tracks recorded through the web browser. The implementation is described in greater detail later in the manuscript (Sect. 4.1.4) and specifically represents a novel contribution compared to the functionalities available in previously reported systems. Figure 2 illustrates the main controls of the Composition view including the latency test option at the top bar.

### Data models

A key consideration in the system design is the definition and organization of data with respect to its intended use. Accordingly, the database schema and entity relationships were structured with reference to established vocabularies and concepts from music production, ensuring consistent and meaningful representation of the information managed by the application.

One of the key ontologies used as a reference for naming database fields was the Music Ontology [31]. This was further complemented by definitions provided in Schema.org [32]. Terms such as Composition and Track—present in both vocabularies—were adopted, though they exhibit slight differences in meaning. In Music Ontology, the term Track typically refers to an album track (a music Composition), rather than an audio track in a DAW environment.

**Fig. 3:**
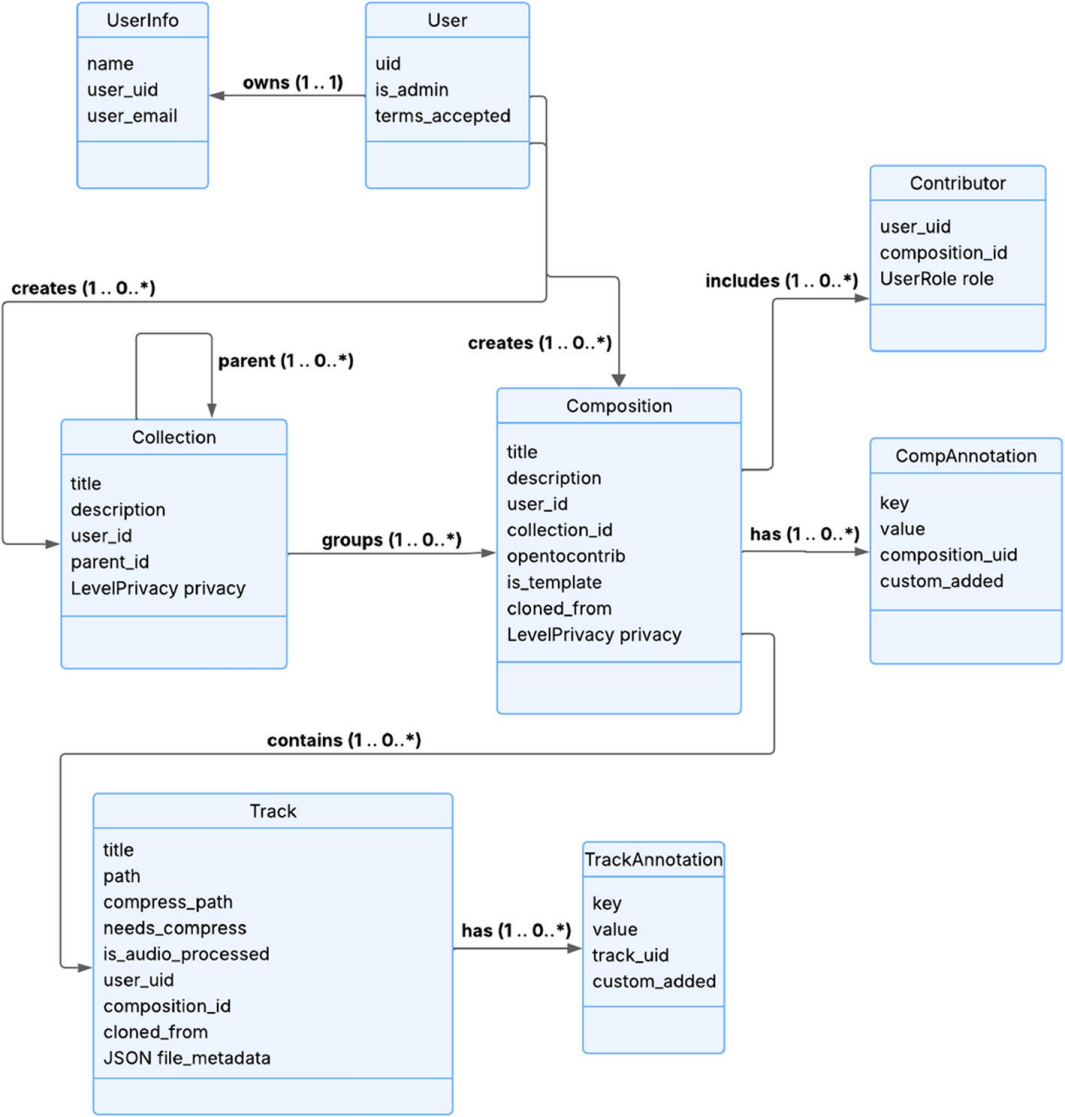
The figure illustrates the different classes used to define the system, as well as to organize and structure the data. Each class is represented by a box, with the main classes being User, Collection, Composition, and Track. Within each box, the properties that characterize each class are listed, serving as links to adjacent classes. The arrows indicate the relationships and dependencies between the classes

From a semantic perspective, the platform builds upon established music knowledge representations, notably the Music Ontology, to support annotation and data organization. Recent ontology frameworks such as the Polifonia ontology network [33] or the pioneer Studio Ontology Framework [34] illustrate how richer semantic models can support musical heritage, production workflows, and interoperability. While these approaches are not directly integrated in the current implementation, the underlying data model of Hi-Audio is designed to remain compatible with such semantic infrastructures. Table 2 summarizes the principal classes defined to organize and structure information within the system, while the diagram at Fig. 3 illustrates the hierarchy and relationships among these classes.

**Table 2:** The main classes defined to structure information in Hi-Audio are listed below, along with a brief description of their meaning

Class/entity	Description
User	The user is the entity responsible for creating data and information within the system. A user account is established using an email address
UserRole	Within a composition, the role defines the permissions assigned to each user regarding the creation or modification of existing data
UserInfo	Aggregates identifying information about the user, such as user ID, name, and email address
Collection	A collection is a container that groups one or more compositions and may also include other nested collections
Composition	A composition represents the combination of one or more audio tracks belonging to the same musical work
LevelPrivacy	The privacy level determines the visibility of a composition, allowing for access control or restriction
Track	An audio track refers to an audio file within a composition. It may capture a single musical instrument or serve as a guide track containing multiple instruments, used as a reference for recording additional tracks
TrackAnnotation/CompAnnotation	A track or a composition annotation stores key-value formatted information about various aspects of an audio track or a composition, including both descriptive content (e.g., instrument, genre) and the annotation’s semantic type
Contributor	A contributor is a user who has been added to a composition by the Holder or an Owner and is assigned a specific collaborative role

### Collaboration, authentication, roles, and privacy

Collaboration enables remote and asynchronous contributions to a single composition, allowing multiple users to collectively build a musical piece by adding individual audio recordings. This process resembles traditional studio-based music production in that it follows a largely sequential workflow. However, Hi-Audio facilitates the geographical and temporal decoupling of participants, enabling contributions to take place at different times and from different locations. Collaboration is supported through user identification via email, which enables role assignment and the definition of privacy levels and access permissions, thereby providing fine-grained control over each participant’s contributions.

Five hierarchical user roles are supported, each associated with distinct permission levels. *Holders* are the original creators of a composition and retain full control over it. *Owners* may be invited by *Holders* and have equivalent permissions regarding audio content and metadata management; however, they cannot delete the composition itself. *Administrators* can manage collaborative content by creating or deleting tracks and annotations added by other users, but they cannot modify composition-level metadata or configuration settings such as collaborator management. *Members* are permitted to contribute audio tracks and edit only the data they have created, without the ability to alter or remove content authored by others. Finally, *Guests* are limited to read-only access and may view composition content without the ability to alter it, which is particularly useful for controlled access to private compositions.

In addition to role-based permissions, each composition is assigned a privacy level that governs its visibility and accessibility. Compositions may be *Public*, allowing unrestricted access to all users, including non-registered visitors; restricted to *Only Registered Users*, limiting visibility to authenticated platform users regardless of collaboration status; or set as *Private*, in which case access is confined to the owner and explicitly designated collaborators.

Although the platform collects minimal personal information—limited to users’ email addresses—it is essential to provide individuals with full control over their data. In particular, adherence to the General Data Protection Regulation (GDPR)^16^ for safeguarding sensitive data when collecting and managing music-related contributions. Beyond ensuring compliance with privacy regulations, the legal framework also addresses intellectual property (IP) concerns through the use of Creative Commons licenses and mechanisms for content ownership and attribution. Together, these measures maintain legal conformity and support the responsible development of distributed music content.

### Collections and annotations

In datasets commonly studied in MIR, as well as in other research domains, hierarchical file organization structures are frequently encountered. For instance, these resources may be categorized based on their purpose—such as training or testing sets. In MoisesDB [35], as another example, compositions are stored within folders, which in turn contain subfolders that group tracks by instrument. This form of organization, typical in the creation of scientific databases, underpins the rationale for the existence of collections in the Hi-Audio system. Collections serve as a flexible mechanism for grouping compositions under a common criterion or nested sub-criteria. They can represent either a conventional music album or a structured subset intended for training a machine learning model.

Annotations are another common feature of scientific datasets. Within the Hi-Audio system, annotations refer to supplementary information attached to audio content, providing specific knowledge and descriptive context about the data. Annotations enhance datasets by enabling more effective data search and retrieval [36], offering the contextual details necessary for meaningful queries. They are also crucial for the development of learning models, particularly in supervised approaches, where labeled data helps models learn to identify, classify, and infer from the content. This is especially pertinent in the audio domain, where sound perception and classification depend on various nuanced criteria—such as timbre, articulation, pitch, or musical notes—many of which are not easily discernible to the human ear and must be explicitly described through annotation.

The system distinguishes between two types of annotations:

Manual annotations: These are created voluntarily by users and typically consist of key-value pairs that may represent either objective or subjective labels. The key defines a concept, while the value assigns its corresponding meaning. For example, in JSON format: {“Inverse Repeat Sequencesperformer”: “John Doe”}. The system provides a set of default manual annotation fields applicable to all audio tracks: title (track name), performer (interpreter), comment, recorded_at (recording location), and recording_date (timestamp).
Automatic annotations: These are generated by algorithms responsible for processing and analyzing audio files. They follow the same key-value format as manual annotations, but are used exclusively to describe objective musical features, such as key, tempo, or sound intensity level (e.g., RMS^17^). Additionally, automatic annotations include metadata extraction from the audio files. Further details regarding the automatic labeling process and its implementation can be found in Sect. 4.2.1.

### Recording scheme and usage scenarios

One of the key features of the application is its ability to easily import existing musical collections and compositions into the database via the web interface. In addition to this, as previously mentioned, users have the option to create audio files directly within the application using any device equipped with a web browser that supports audio capture from an input source, such as a microphone.

Alternative recording methods involving line-in instrument inputs—such as electric guitars or keyboards connected directly to an audio interface—are not recommended, as they may pose challenges in accurately estimating latency. Proper operation in such cases depends on the audio interface in use, particularly whether it supports input monitoring or loopback functionality. For MIDI sources, compatibility is not guaranteed, as the system does not natively support the Web MIDI API. To address these limitations, the system documentation explicitly communicates the relevant constraints and usage conditions to users. Further technical details, including the operating systems, browsers, and respective versions used during development and ad hoc testing, are provided in the Hi-Audio support page.^18^

A general best practice when recording digital audio in multitrack compositions using a DAW is to use wired headphones instead of Bluetooth-connected ones. Bluetooth headphones, even those with built-in microphones, are generally discouraged due to the high and variant latency they introduce, which is difficult to estimate and compensate for accurately. In addition, the audio capture codecs used in Bluetooth devices are often optimized for voice transmission rather than music, leading to significant degradation in input signal quality resulting from limited spectral fidelity.

Beyond the general workflow illustrated in Fig. 1, a typical procedure for a registered musician to record and save a multitrack composition consists of three main steps. First, a composition is created either independently or within a collection, with users providing basic metadata such as title, description, and privacy settings. Second, a built-in tool supports estimation of browser round-trip latency to account for hardware- and software-induced delays during recording (see Sect. 4.1.4). Finally, users may optionally employ additional utilities available in the composition view, including a metronome for temporal reference and a microphone test for input level verification, depending on the recording configuration.

The intended usage scenarios supported by the developed software are based on the concept of a guide track [37]. A guide track is a temporary audio recording used in a DAW session to provide a reference for other musicians/tracks during the recording process. It helps establish the tempo, pitch, structure, and feel of the song. It can be as simple as a vocal melody, a rhythm guitar, or a basic arrangement of multiple instruments. Guide tracks help to continue or expand the work within a composition. Based on this model, the following usage modes can be established, each supporting different privacy levels, depending on the user’s preference for public exposure:

Single-user composition without roles: This default usage mode is analogous to that of a traditional desktop DAW. A user can import or record audio files sequentially to build the composition without involving other remote collaborators.
Collaboration by invitation: The composition holder or owners can invite one or more participants by email and assign roles according to the level of permissions they wish to grant, as described in Sect. 3.2.
Open to contribution: In this scenario, any registered platform user can contribute voluntarily without requiring an invitation. For this to be possible, the composition must not be private—only compositions marked as public or visible to registered users are eligible. It is important to note that tracks uploaded by contributors are not subject to any review process, which may result in the publication of undesired content under a single composition without explicit consent.
Clone: Within the composition settings, there is an option to mark a composition as a template. When a composition is designated as a template, it becomes available for any user to copy and reuse its content to create a new version based on the original (i.e., remix or derivative work). As a real-world example, this method was used to create the Happy Birthday collection^19^. The base version served to produce different renditions in multiple languages and with various instruments.

The recording scheme and usage scenarios described in this section also informed the design of the user evaluation presented in Sect. 5.2. In particular, the study tasks were defined to reflect the intended recording workflow, recommended usage constraints, and collaboration modes supported by the platform, thereby serving as a proof of concept of the proposed interaction model under realistic conditions. This alignment ensures that the evaluation assesses not only isolated functionalities but also the practical feasibility of the system’s recording and collaboration framework as a whole.

## Implementation

The system has been implemented following a client–server application model, a widely adopted architecture in the design of internet-based services. This approach enables centralized management of resources, which is essential for supporting asynchronous and remote user interaction. Based on the chosen paradigm for information exchange, the system is structured into two distinct layers: client and server, each of which is detailed in this section.

**Fig. 4:**
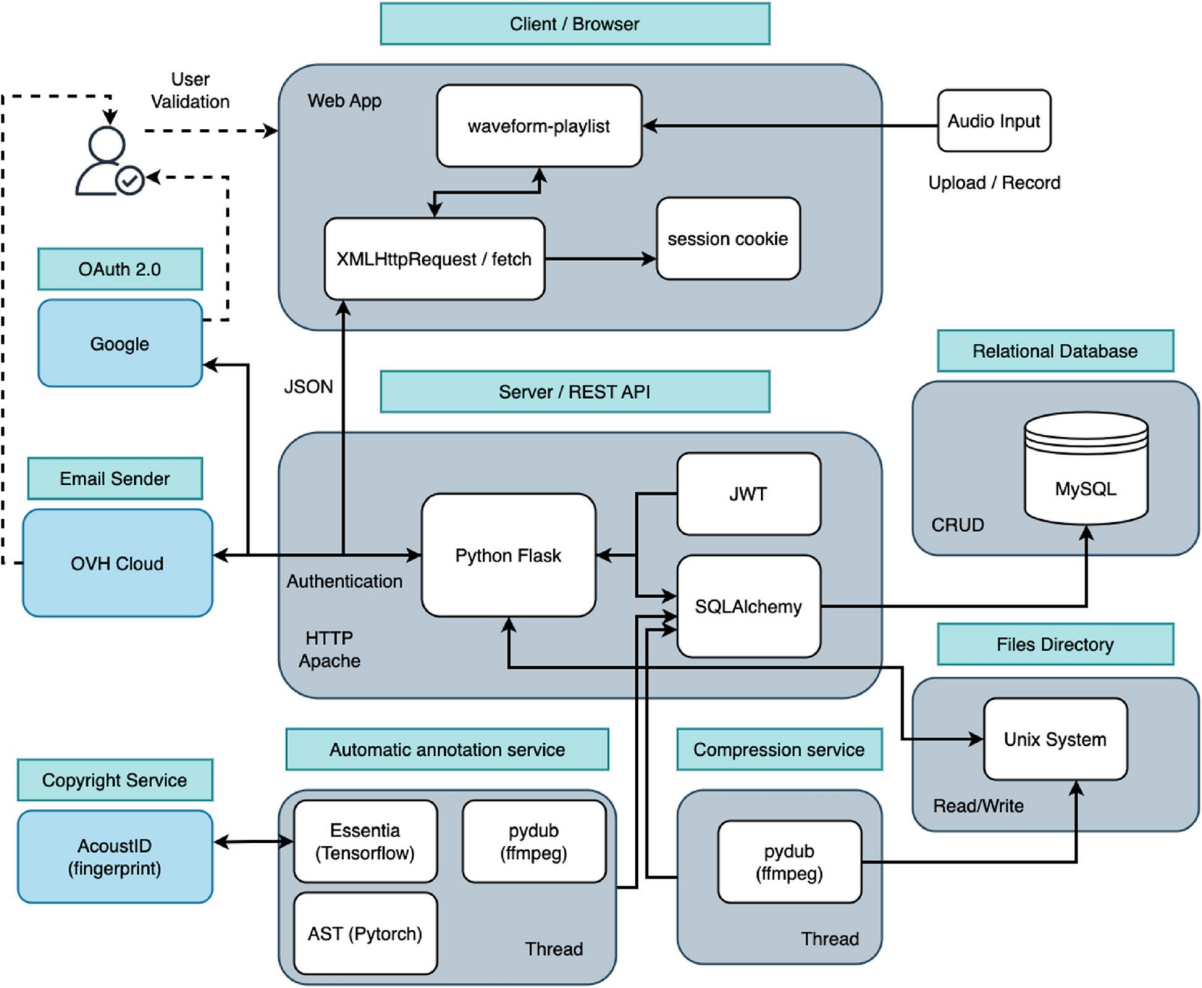
The diagram illustrates the various components and services involved in the system’s operation. The web application interacts with a dedicated server for data management. External providers, such as Google and OVH Cloud, are utilized for user authentication. The back-end employs two separate threads for data annotation and audio compression, respectively. Additionally, the AcoustID service is queried to identify copyrighted tracks in the database using audio fingerprinting

The following subsections specify the various programming languages, technologies, and frameworks employed during implementation, along with key design decisions made to ensure system reliability and performance. The high-level architecture diagram represented in the Fig. 4 illustrates the main components of each module.

### Client

For the client-side (or front-end) implementation, a web application was developed using standard technologies: JavaScript, HTML, and CSS. Other services mentioned earlier—such as Soundtrap and BandLab—offer native applications for Android and iOS in addition to their web versions. These native apps generally provide a superior user experience by not being constrained by browser limitations. However, developing and maintaining native applications incurs higher costs due to the need to support multiple operating systems.

Web applications offer several advantages over native apps. The primary benefit is the universality and reusability of the code across platforms. A web application can run on various operating systems without requiring additional installation, aside from a web browser, which is typically available by default. In contrast, desktop or mobile applications require maintaining separate versions in different programming languages for each platform.

Another advantage of web applications is that users always access the most up-to-date version of the software, as the latest code is delivered directly to the client. In native applications, users must manually install updates. This versatility—combined with the advanced capabilities of modern browsers via the Web Audio API—makes a web application a technically viable and efficient alternative to a native app in the context of this study. The main limitation of web applications compared to installable software is their dependency on an internet connection for execution, storage, and synchronization of data.

An additional constraint in web client development stems from the inconsistent implementation of the Web Audio API across different browsers and versions. This issue is known as fragmentation in the Android ecosystem and arises in all operating systems due to varied adoption rates of software versions. Specifically in Android, fragmentation refers to version disparities across manufacturers, but the term is equally applicable to inconsistencies in Web Audio API support across browsers. In certain cases, polyfills^20^ or browser-specific prefixes must be used to ensure compatibility.

The client-side implementation in Hi-Audio utilizes several third-party open-source libraries, most notably Bootstrap^21^ (v4.6) and waveform-playlist^22^ (v4.3.3). Bootstrap is a widely used front-end toolkit that provides design templates for forms, buttons, navigation menus, and other responsive UI (User Interface) components. It facilitates consistent visual presentation across different devices and abstracts away many compatibility issues associated with supporting multiple browsers from a single codebase. Additionally, Bootstrap is a dependency of waveform-playlist, the core library used for implementing the multitrack editor.

The decision to adopt waveform-playlist over other libraries such as Wavesurfer.js^23^ was informed by prior work in the field of online DAW development [38–40]. Unlike Wavesurfer, waveform-playlist offers integrated support for multitrack recording, playback, and waveform visualization by interacting directly with the Web Audio API. This library uses the WAV format for recording audio and for exporting final mixes from the AudioBuffer.

Integrating waveform-playlist into the Hi-Audio platform required several custom modifications to meet specific system requirements, such as latency compensation (see Sect. 4.1.4). To support these changes, an independent fork of the library was created on GitHub^24^, where improvements, issues, and pull requests are documented and maintained.

#### Import script

To facilitate the automated import of existing musical datasets and avoid the tedious task of manually uploading files to the server, a script has been developed as part of a separate repository^25^. This script, which is currently under development, is written in Python and interacts with the Hi-Audio API using the authentication token. It allows users to specify, via the command line, the path to an input directory containing musical datasets (folders and audio files) and performs synchronization with the server while preserving the original subdirectory structure and hierarchy.

The import process results in:

1. The creation of Collections for folders that contain other sub-folders, and
2. The creation of Compositions for folders that contain only audio tracks.

The script is designed to streamline the publication of existing content and serves as a general-purpose tool. However, it may require adjustments based on the specific characteristics of the datasets being processed, particularly for the inclusion or exclusion of certain files or information present in the directory. While not all datasets share the same structure in terms of metadata and file organization, commonalities exist that form a foundation for automation.

For some of the tests conducted with the import script (see Sect. 4.2.1), the music database DSD100 [41] was loaded. The DSD100 is a dataset of one hundred full lengths music tracks of different styles along with their isolated drums, bass, vocals and others stems (e.g., piano and guitar). DSD100 contains two folders, a folder with a training set: "train", composed of 50 songs, and a folder with a test set: “test”, composed of 50 songs. The following listing illustrates the output of the tree command-line utility applied to the DSD100 subset, showing the structure of the folders, which contains only four songs. 
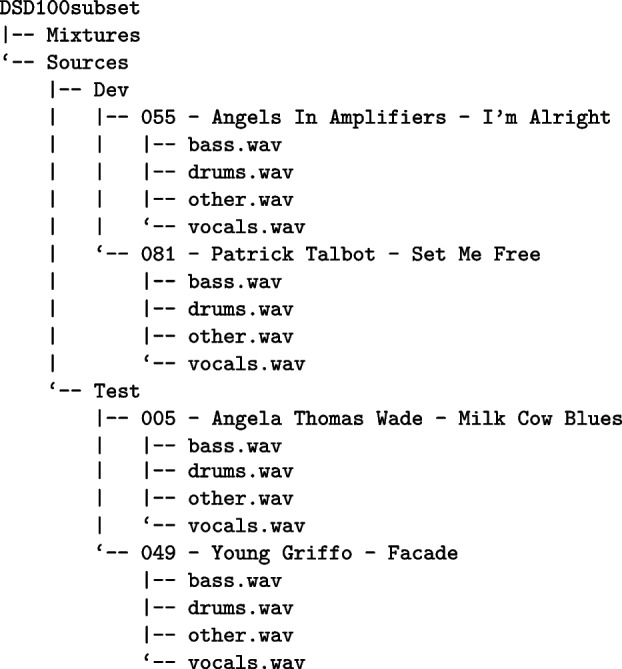


It is worth noting that the communication interface between the client and server—whether for the import script or the web application—follows a RESTful architecture [42]. Operations are performed via HTTP using JSON-formatted messages.

#### Audio constraints

One of the necessary modifications made to the original waveform-playlist code to enhance audio recording quality involved adjustments to the so-called audio constraints. Audio constraints imposed by the browser can be controlled from the JavaScript code. When using the Web Audio API, applying certain constraints can introduce signal filtering operations that degrade input audio quality due to built-in processing aimed at eliminating echo, background noise, and other artifacts.

These constraints are defined through a set of parameters applied when configuring the incoming audio stream using the getUserMedia method, which is responsible for accessing the microphone. Constraints allow developers to disable default browser settings such as automatic gain control and may operate at two levels: browser-level and device-level, both of which may affect the quality of the captured audio signal. The following example shows how constraints and the getUserMedia method are used in Hi-Audio, with boolean variables explicitly set to false:
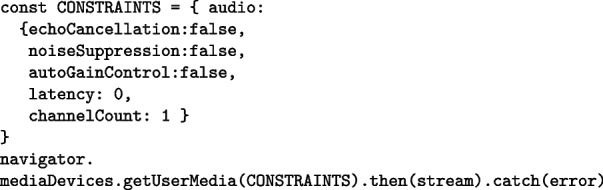


One of the main reasons for disabling these constraints is the requirement to perform latency estimation, which necessitates recording a signal emitted from the audio output. If echo cancellation is active, it will suppress the recording of the sound being played, making accurate measurement impossible. In fact, the latency compensation technique described in Sect. 4.1.4 relies on the emission of a specific pseudo-random noise—which may be adversely affected if the noiseSuppression constraint is enabled.

The latency constraint [43], specified in seconds, controls the time elapsed from the initiation of audio capture (i.e., when a sound occurs in the real world) until the data becomes available for further processing. While higher latency may be acceptable in certain applications as a means to reduce power consumption, the value set in Hi-Audio is 0 by default. This value signals to the browser that the lowest possible latency is desired, prompting the system to minimize it as much as the environment allows.

#### Memory limitations

Regarding memory limitations, specific issues have been observed during ad hoc testing when multiple tracks are loaded in a composition. The memory demand increases due to the use of the decodeAudioData [44] function within the waveform-playlist library. In particular, excessive memory consumption errors may occur on devices with limited hardware capabilities, such as certain mobile phones, for instance, in Firefox [45] on Android. The memory demand increases proportionally and may exceed available resources, leading to failure or degraded performance. decodeAudioData operates only on complete audio files which makes it inherently slow and memory-intensive, especially for large files.

Some browsers, such as Chrome and Safari, have optimized implementations of this method, which help reduce the performance impact and memory load during audio file decoding. Currently, several alternatives exist such as (1) audio-file-decoder^26^, (2) decode-audio-data-fast^27^ or (3) wasm-audio-decoders^28^, to mitigate this issue within the client-side code; however, none have been implemented or further explored at this stage. Nevertheless, since this limitation stems from the inherent behavior of the Web Audio API, it represents a noteworthy consideration for future development and is thus documented in this section.

#### Round-trip latency estimation

Latency, referred to here as round-trip latency, is an inherent phenomenon in audio capture and restitution that adversely affects the temporal alignment of audio tracks. Even when minimal, it manifests as an undesired delay between the audio input and the corresponding output. Accurate latency estimation when working with digital audio equipment is critical for the correct operation of certain applications, particularly DAWs and other tools used in music creation and editing. These systems require precise synchronization, which is essential for reliable recording and mixing processes.

In the context of the Hi-Audio online platform, client-side audio round-trip latency is considered, defined as the time elapsed between an audio signal entering the local audio input (e.g., microphone), being processed by the browser’s audio pipeline, and being rendered back at the local audio output (e.g., headphones or loudspeakers). This measurement characterizes the latency introduced by the operating system, audio drivers, hardware, and the Web Audio API execution within the client’s browser.

**Fig. 5:**
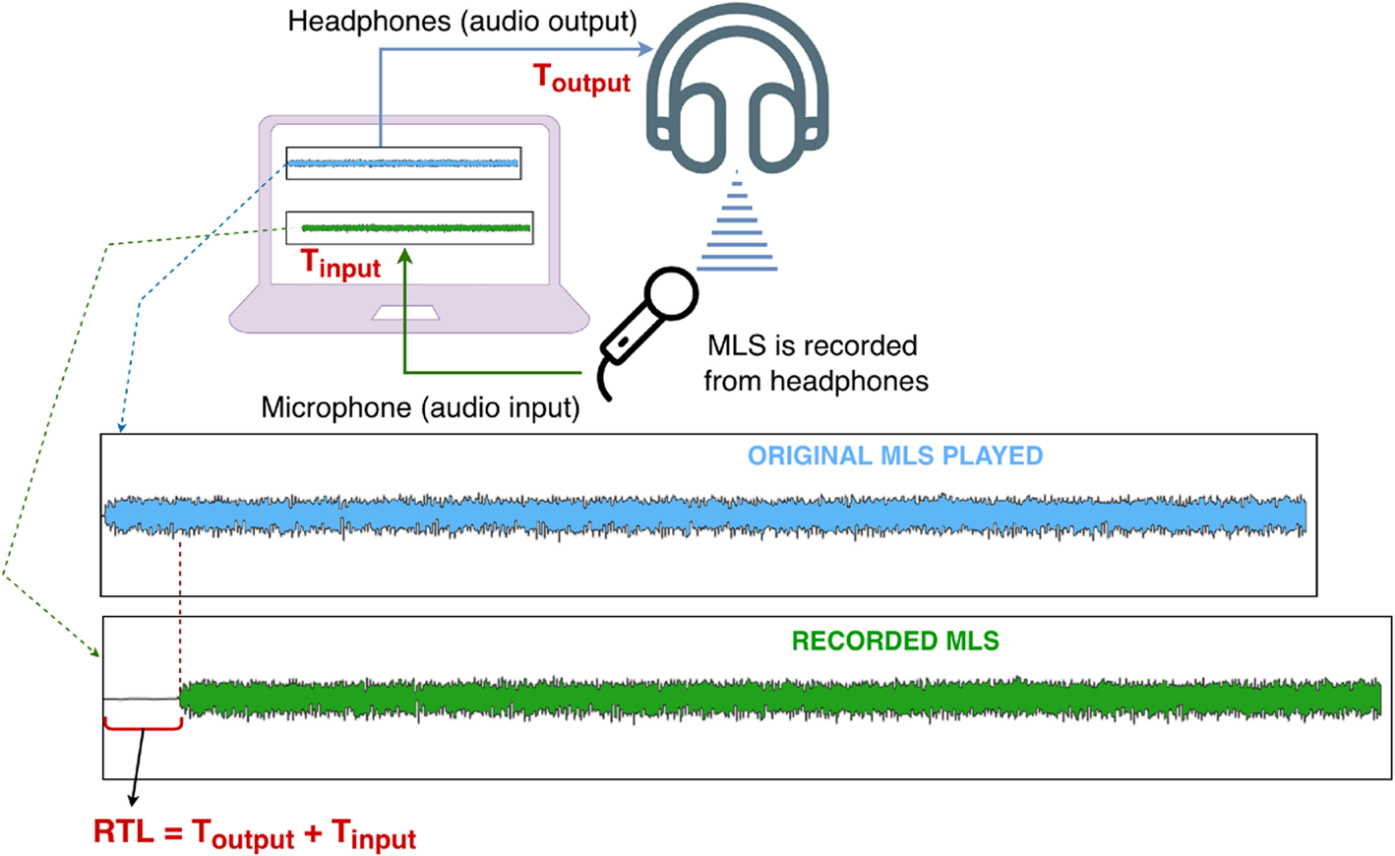
Diagram illustrating the procedure used for round-trip latency (RTL) estimation based on a MLS sequence. The MLS signal is reproduced through the headphones and simultaneously captured by a microphone. The measured RTL corresponds to the sum of the input and output latencies, $T_{\textrm{input}} + T_{\textrm{output}}$

As previously outlined, recording a new track in the proposed framework based on guide tracks, entails musician performing directly onto an existing audio file. In this context, a critical challenge is the accurate estimation of the round-trip latency associated with the current recording session [46, 47]. The estimation procedure typically consists of capturing the system’s audio output (e.g., loudspeakers or headphones) using the input device (e.g., microphone), assuming that both are co-located, see Fig. 5.

Importantly, the latency considered here does not include any contribution from network transmission or server-side processing. Network latency is inherently variable, highly dependent on external factors such as network conditions, routing, and geographical distance. The primary objective of the latency estimation mechanism is to enable accurate temporal alignment between a newly recorded track and the reference. Without compensating for round-trip latency, the recorded signal is systematically shifted relative to the guide track, resulting in perceptible timing misalignments. Since audio playback and capture in the Web Audio API rely on local buffering and processing within the browser, this latency depends exclusively on the client’s hardware, operating system, drivers, and browser implementation, and is therefore independent of network transmission conditions.

By focusing on browser-level round-trip latency, the platform enables musicians to better understand and compensate for systematic timing offsets during recording. This design choice is particularly relevant for asynchronous collaboration scenarios, where recordings are captured locally and synchronized offline rather than streamed in real time between participants. Consequently, the latency estimation serves as a calibration aid for dataset creation.

Round-trip latency can be expressed as the sum of the input and output latency [48]. In theory, audio input and output latencies are constant; however, in practice they may vary significantly over time due to system adaptations to environmental and operational conditions, such as energy-saving mechanisms or noise-dependent processing.

Input latency is dependent on the audio buffer size used and is available through the AudioContext baseLatency property. Output latency is defined as the time interval between the onset of audio processing and the actual sound playback and can be analytically estimated via the AudioContext outputLatency property [49]. While such properties may provide insight into browser behavior in specific scenarios, they cannot serve as a reference for compensating round-trip latency during recording, which instead requires direct audio measurements.

For the Hi-Audio platform, a novel and more robust approach has been adopted for latency estimation, based on the use of maximum length sequences (MLS) [50] as illustrated in Fig. 5. Conventional approaches typically rely on emitting predefined acoustic signals, such as impulsive clicks or sinusoidal beeps [51–53]. However, their reliability degrades in acoustically adverse environments with background noise or reverberation, and sinusoidal beeps may be perceptually intrusive. In contrast, MLS are pseudo-random noise sequences characterized by an auto-correlation function that closely approximates a Kronecker delta, enabling accurate estimation of round-trip delay [54]. These periodic sequences, originally generated via maximal linear-feedback shift registers, were first introduced by Schroeder in 1979 for room impulse response (RIR) measurements [55].

Despite their utility, MLS-based methods are known to exhibit certain limitations, including time-aliasing and distortion artifacts. Time-aliasing arises during the auto-correlation computation when the sequence length $L$ is insufficient relative to the duration of the room impulse response to be measured. However, this limitation is not critical in the present context, as the objective is solely to measure the primary latency, and sufficiently long sequences are employed. In the case of study a $0.75$-s MLS is used, offering an effective trade-off between computational complexity and estimation accuracy.

Another well-documented drawback of MLS is the presence of spurious peaks—referred to as distortion peaks—introduced by nonlinearities in the measurement system. To mitigate these artifacts, alternative sequences such as inverse repeat sequences (IRS) have been proposed [56]. Nonetheless, in the Hi-Audio application, such distortions are not detrimental, given that the focus is restricted to estimating the dominant delay component, rather than reconstructing a detailed RIR.

The confidence of the round-trip delay estimation is assessed by computing the following ratio [50]:

1 $$\begin{aligned} R=10 \log _{10} \left( \frac{max_\tau [C(\tau )^2] }{\frac{1}{N} \sum _1^N C(\tau )^2} \right) \end{aligned}$$

where $C(\tau )$ is the cross-correlation between the input and output signals at time lag $\tau$ and $N$ is the number of samples of the cross-correlation (typically $N=22050$ corresponding to $0.5\,s$ for signals at $44.1\,kHz$). In practice, a predefined fixed threshold set to $+18dB$ is defined to consider the test successful.

The variability of the round-trip latency measured across different web browsers and operating systems was studied in [50]. The results, reported in Table 3, were obtained by executing the latency measurement application—based on MLS and integrated into the Hi-Audio platform—100 consecutive times^29^. Among the tested browsers, Firefox demonstrated the most stable performance, with the standard deviation (SD) frequently being zero or near-zero. This indicates that the latency values remained consistent across trials, which is a desirable characteristic for achieving accurate delay compensation. In contrast, Chromium-based browsers, such as Chrome and Edge, exhibited significantly greater variability, with SD around 8 ms, particularly on systems such as Ubuntu, and Windows.

**Table 3:** Round-trip latency results over 100 consecutive tests for different browsers and systems. The table reports the mean latency, standard deviation, and the corresponding minimum and maximum values, all expressed in milliseconds (ms), from [50]

System/browser	Mean (ms)	SD (ms)	Min (ms)	Max (ms)
**HP Ubuntu**				
Chrome	64.50	7.94	49.37	85.17
Chromium	64.15	8.21	41.41	76.44
Firefox	65.69	0.00	65.69	65.69
**Lenovo Windows**				
Edge	60.82	6.06	55.23	96.00
Chrome	62.84	2.44	61.42	73.42
Firefox	104.65	0.00	104.65	104.65
**MacBook Pro 2021**				
Safari	100.02	0.00	100.02	100.02
Chrome	52.33	1.14	49.98	52.88
Firefox	38.89	0.00	38.89	38.89

The proposed approach was extensively evaluated under a wide range of recording conditions, including acoustically challenging and high-noise environments, using diverse hardware configurations—such as built-in microphones and loudspeakers, line-in instruments with external sound cards, and Bluetooth headsets—and across multiple web browsers. Overall, the method demonstrated strong robustness and efficiency, particularly when compared to conventional latency estimation techniques based on impulsive noise clicks or sinusoidal test signals. Nevertheless, the results reported in Table 3 reveal a key remaining challenge for round-trip latency estimation, namely the heterogeneity of hardware platforms, web browsers, and underlying audio processing technologies, which limits consistency across settings. In particular, the presence or absence of embedded audio processing features, such as echo cancellation or noise suppression, can alter the playback or capture of the MLS sequence and compromise delay estimation accuracy. Additionally, the use of hands-free Bluetooth headsets remains an unresolved limitation, as their reliance on distinct audio transmission protocols and lossy compression can significantly degrade the MLS signal, thereby impairing its effectiveness for latency measurement.

### Server

Regarding server-side technology, or the back-end, the implementation is driven by the selection of Python as the primary programming language. This decision is justified by the need for robust tools and methods for audio file processing, particularly for the machine learning techniques used in automatic labeling. Python has become the de facto language for AI applications in recent years, owing to its rich and well-supported ecosystem.

Furthermore, Python provides web frameworks in the form of packages and modules that simplify the development of application programs [57]. They support both server-side and client-side programming by handling several key activities, such as request interpretation (e.g., retrieving form parameters, managing cookies and sessions), response generation (e.g., producing output in HTML or formats like JSON), and data storage. Most frameworks include templating and data persistence as essential components for building web applications. Data persistence involves storing and retrieving data while ensuring consistency.

As described in Sect. 4.1.1, the server implements a RESTful API using the Flask framework^30^. The database system employed is MySQL, and database management is handled through SQLAlchemy, an open-source Python library. SQLAlchemy offers a comprehensive set of SQL tools and an Object-Relational Mapper (ORM) that enables efficient interaction with the database using Python objects, providing both abstraction and flexibility.

The following subsections describe the considerations for implementing the automatic labeling and audio file compression modules on the server.

#### Automatic track annotation and metadata extraction

The system includes a dedicated processing thread responsible for handling incoming audio files on the server and performing automatic annotation [58] or labeling of tracks, complementing the manual annotations provided by users. The libraries used for audio analysis and automatic annotation are (1) Essentia [59], for music and audio analysis and (2) AST [60], for instruments recognition:

1. Essentia is an open-source library written in C++ with Python bindings. It is designed for audio analysis and music information retrieval, offering a broad range of algorithms for digital signal processing, high-level musical descriptors, and deep learning-based inference tools. Essentia provides a wrapper for TensorFlow [61] that allows using virtually any TensorFlow model within its audio analysis framework.
2. AST (Audio Spectrogram Transformer), implemented using PyTorch^31^, is a convolution-free, purely attention-based open-source model for audio classification which supports variable length input and can be applied to various tasks. It applies a Vision Transformer to audio, by turning audio into an image (spectrogram). The model includes an audio classification head on top (a linear layer on the pooled output), which is used for tasks such as classification on datasets like AudioSet [62] and Speech Commands v2.

In selecting a suitable model for musical instrument recognition, several tests were conducted using the Import script (Sect. 4.1.1). The evaluated models included those available in Essentia, which were compared with more recent approaches such as AST. The latter yielded better results, which led to its inclusion in the system for the automatic annotation of the instrument label. To verify the model’s performance, several scripts—available on GitHub^32^—were created to analyze the DSD100 (see Sect. 4.1.1) and MoisesDB datasets. These scripts are employed to compute the accuracy of the AST model in the instrument recognition task. The program runs through each dataset and compare the proposed label for each audio track with the model’s output. To obtain consistent results, similar label sets were mapped to match the existing values in the target dataset, thereby enabling comparison. In the case of DSD100, (see Sect. 4.1.1) only the labels “bass,” “vocals,” and “drums” were used (the label “other” was omitted), and the corresponding groups were created as shown below.
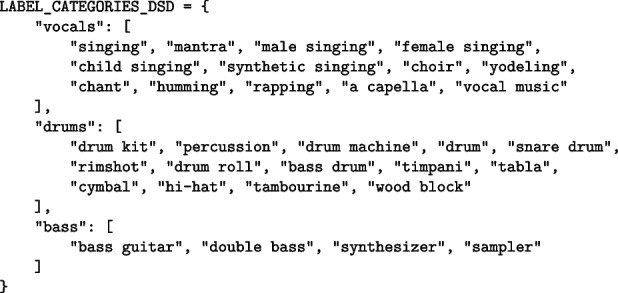


The same approach was applied to MoisesDB by defining a dedicated dictionary that maps label variants to their corresponding instrument categories. The analysis performed one-class classification over the complete datasets. The model achieved an accuracy of 92.00% on the DSD100 dataset, correctly matching 276 out of 300 tracks. For MoisesDB, the model obtained an accuracy of 87.29%, corresponding to 2219 correct predictions out of 2542 tracks. These results indicate a consistently high recognition performance across both datasets, despite differences in size, annotation schemes, and label variability.

**Fig. 6:**
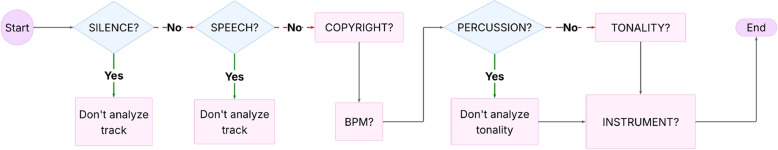
Diagram displaying the decision tree implemented at backend level for audio processing and automatic labeling

Since the automatic annotation task is executed server-side, particular care has been taken to minimize computational cost in order to reduce queue wait times for file processing and to avoid unnecessary annotation of tracks. To ensure efficient management and reduce processing time, a hierarchical labeling structure is employed through a decision tree. This allows conditional analysis based on whether a track meets certain criteria or exhibits specific characteristics. For instance, the system first attempts to estimate the sound level before proceeding to content prediction. If it determines that the track consists mostly—or entirely—of silence, no further annotation is applied. The decision-making process followed by the annotation algorithm is represented in the decision tree at Fig. 6:

The extracted information is presented to users through the web interface in the form of a modal dialog associated with each individual track. This dialog allows users to view, edit, and manage annotation metadata as key–value pairs, thereby enabling direct interaction with and refinement of the information generated by the platform. Among the most relevant automatic labels that are produced within the system, the following can be found:

- is_silence: is set to true or false based on the RMS value measured in decibels (dB). A threshold of – 48 dB has been defined to distinguish silent audio.
- is_human_voice: if the probability obtained for the speech label (see Fig. 6) is higher than 8%, the content of the track is probably a human voice either singing or speaking and then is labeled as true. It is only cataloged as speech if the probability is higher than 50%.
- is_copyrighted: in case of match when querying the AcoustID^33^ service (see diagram Fig. 4) with the track fingerprint, a percentage is retrieved, telling the chances of the track to be copyrighted. The title and the artist information can be included too.
- BPM: in modern Western music, tempo (the speed of a piece) is usually indicated in beats per minute (BPM). This means that a particular note value (for example, a quarter note) is specified as the beat, and that the amount of time between successive beats is a specified fraction of a minute [63].
- is_percussion: it is a boolean tag. It is set to true in case the Essentia model [64] for music loop instrument role classification finds the class percussion.
- tonality: tonality refers to the hierarchical system of pitches centered around a tonic note. Tonality includes features such as key signature (main note) and scale (major or minor) [65].
- instrument: consists of two parameters, the score and a label, that are obtained using the AST model telling the predicted instrument.

Alongside the annotations, a set of metadata is also collected for all audio tracks registered in the database. These metadata provide information related to the audio file format, including: bit rate, channel layout (mono or stereo), codec name, sample rate, duration, etc. Metadata are extracted using the Python library pydub^34^.

#### Server-side compression and audio formats

When working with audio files for musical compositions on online platforms, two main challenges typically arise (1) compatibility of audio formats across different web browsers, and (2) the large file sizes produced, particularly when high-quality, lossless formats such as WAV are used.

WAV is the format employed on the client side via the waveform-playlist library to capture audio input samples. Depending on parameters such as sampling rate and bit depth, file sizes can vary significantly. In general, WAV is the most widely used format in existing multitrack datasets within the literature. A typical configuration uses a sampling rate of 44.1 kHz and 16 bits per sample. Under this configuration, a 3-min audio track may occupy approximately 35 MB. Consequently, a composition containing four tracks can result in a total size of around 120 MB—an amount that can pose considerable challenges for upload and download, particularly over constrained network connections such as 3G, 4G or areas with limited coverage.

Regarding audio file uploads from the client, there are limited options to improve upload speed, as maintaining the original recording quality is a priority. Therefore, lossy compression during upload is not appropriate. However, to improve the download experience—particularly from a user interface and network efficiency standpoint—a server-side mechanism has been implemented for file conversion and compression. This process runs asynchronously in the background, independent of the main workflow, and targets heavier file types such as WAV and FLAC. These are automatically converted to AAC (Advanced Audio Coding) [66], a lossy format developed as a successor to MP3 that offers superior audio quality at lower bitrates.

Known limitations have been identified regarding audio format compatibility across browsers and operating systems. Compatibility typically depends on both the browser and the underlying system, often requiring the presence of appropriate codecs. For instance, support for AAC in Firefox may require additional codecs on certain GNU/Linux distributions. On macOS, a recent system update (version 15.5, Sequoia) has enabled Safari version 18.4 and later to support playback of Ogg Vorbis files, which were previously incompatible^35^. On Chrome, no significant limitations have been observed.

A further complication in audio format compatibility relates to codecs within file containers. In particular, on Apple systems, containers with.m4a extensions—typically associated with AAC—can also include ALAC (Apple Lossless Audio Codec). These files may not be playable in browsers such as Chrome or Firefox on macOS, with Safari being the only compatible option.

The considerations discussed in this section—regarding download speed, data accessibility, and the standardization of various audio formats—highlight the necessity of applying file conversion techniques based on both size and format to ensure proper use and distribution. To address this, the pydub library (referenced in Sect. 4.2.1) has been integrated into the system as the tool responsible for compressing audio files, specifically by converting them to AAC coding. The container or file format is held by the MP4 format [67] (formally MPEG-4 Part 14), using the extension .m4a.

AAC was selected as the target format because, although it is not as universally standardized as MP3, it was designed to deliver superior audio quality at similar file sizes. Original files created by users in WAV or FLAC format are not deleted; they remain stored in the database within the same directory.

## User evaluation

A preliminary user evaluation was conducted to assess the practical usability and applicability of the Hi-Audio online platform from the perspective of amateur musicians. The evaluation complements the technical validation presented in previous sections by examining how end users interact with the system during realistic usage scenarios. Specifically, it seeks to provide empirical evidence in relation to the research contributions formulated in Sect. 1.

### Participants

Participants were recruited through *La Scène*^36^, a student-led music association affiliated with Télécom Paris that received project funding to administer user compensation; as a result, the sample consisted predominantly of engineering students who are amateur musicians, along with a small number of non-student participants. In total, 22 individuals completed the evaluation. The age distribution reflected the predominantly student population: twenty participants were between 18 and 25 years old, one participant was between 26 and 32, and one participant was between 41 and 55. Participants exhibited a strong musical profile, with nearly all reporting prior musical training and regular practice, and many being accustomed to collaborative music-making contexts. Technical background and prior experience with digital audio workstation (DAW) software varied across participants, resulting in a heterogeneous yet representative user population.

**Fig. 7:**
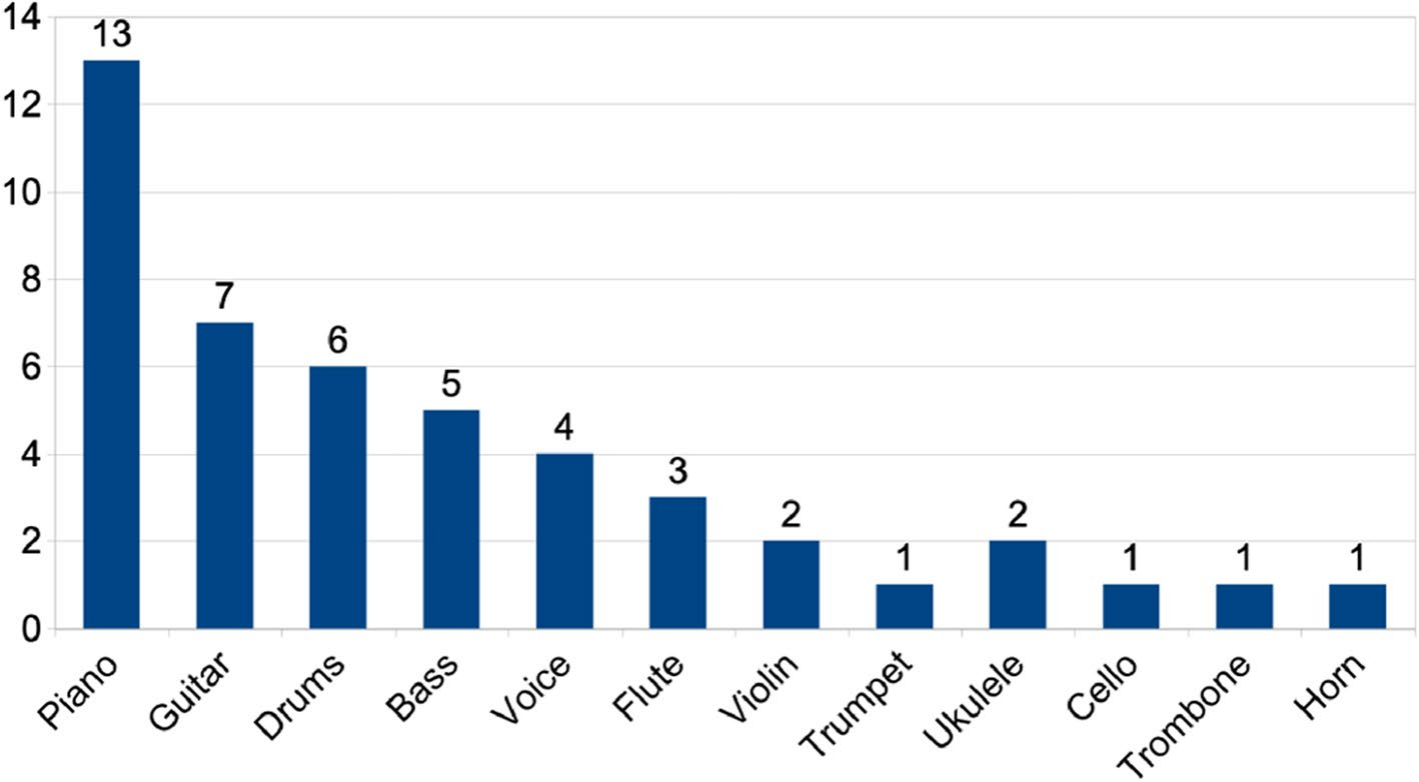
Reported musical instruments bar chart. Participants were allowed to report multiple instruments with independent self-assessed proficiency levels

The participant pool consisted of multi-instrumental musicians. Piano ($n=13$) and guitar ($n=7$) were the most frequently reported instruments, followed by drums ($n=6$), bass ($n=5$), and voice ($n=4$), as shown in Fig. 7.

### Evaluation protocol and procedures

The evaluation was conducted under realistic and largely uncontrolled conditions. Participants used their own devices and audio equipment, and all performed the tasks remotely and without supervision—with the exception of two individuals who completed the test in person at the development team’s facilities. A variety of hardware and software configurations were therefore represented. Although the task instructions recommended the use of the Firefox browser and discouraged Bluetooth audio devices due to potential latency issues, some participants did not follow these guidelines. This variability further increased ecological validity by more closely approximating real-world usage scenarios.

The study comprised two exercises including ten specific tasks covering the platform’s main functionalities, as well as the recording scheme and usage described in Sect. 3.4. The tasks addressed key aspects of the software, including collaborative audio recording, use of recording templates, collection and composition handling, metadata annotation, collaboration management, and estimation of browser round-trip latency.

The task descriptions (see Table 4 for the complete list of tasks) were provided to participants in a document that outlined, in a concise and minimally guided manner, the objective to be achieved at each step of the exercise. This approach was intentionally adopted to rely on users’ intuition when performing the tasks, thereby enabling the identification of usability shortcomings that would be less likely to emerge if more detailed documentation had been provided. Following test completion, participants answered a questionnaire including background and exercise-related questions, the System Usability Scale (SUS) [68], and the NASA Task Load Index (NASA-TLX) [69]. Complete results, including scoring procedures and statistical analyses for both instruments, are provided in Availability of data and materials.

**Table 4:** Task completion rates for the user evaluation, including exercises and tasks numbers

Exercise	Task	Description	Completed (%)	Completed (*n*)
Ex. 1	T1	Record in an existing collaborative composition	68.2	15/22
Ex. 1	T2	Manually annotate track metadata (language)	31.8	7/22
Ex. 1	T3	Update composition title with country/language reference	59.1	13/22
Ex. 1	T4	Select and clone an appropriate recording template	77.3	17/22
Ex. 1	T5	Estimate browser round-trip latency (screenshot)	95.5	21/22
Ex. 1	T6	Record performance on top of existing tracks	90.9	20/22
Ex. 2	T7	Create a new collection	72.7	16/22
Ex. 2	T8	Create a composition within a collection	77.3	17/22
Ex. 2	T9	Record using metronome and/or a guide track	100.0	22/22
Ex. 2	T10	Invite a collaborator via email	45.5	10/22

### Task-based performance results

Table 4 summarizes the task completion rates across all participants. Overall, 72% of the assigned tasks were completed successfully, indicating that most participants were able to achieve the core objectives of the evaluation using the platform.

Tasks directly related to the platform’s primary functionality—audio recording and performance—showed consistently high success rates. Estimation of round-trip latency (T5) was completed successfully by 95.5% of participants, and recording audio on top of existing tracks (T6) reached a success rate of 90.9%. In the second exercise, all participants were able to record a new track using either the built-in metronome or an uploaded guide track (T9).

Lower completion results were observed for tasks related to metadata annotation and collaboration management. Manual annotation of track-level metadata (T2) achieved the lowest success rate (31.8%), followed by T10, inviting collaborators via email (45.5%).

### Questionnaire-based results

The post-task questionnaire provides complementary insight into participant background, execution conditions, and subjective perceptions of usability and workload.

**Fig. 8:**
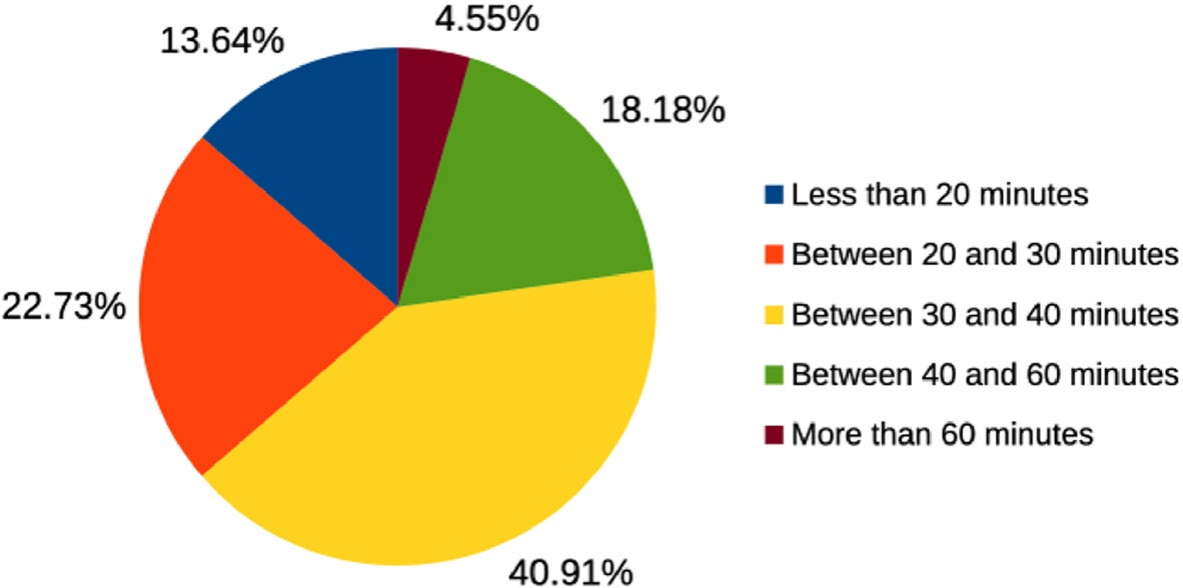
Distribution of self-reported task completion times across participants

All participants reported that this was their first interaction with the Hi-Audio platform. Most completed the tasks within the expected duration of 20–40 min, see Fig. 8, although 4.5% took more than 60 min and 13.6 % took less than 20 min. Approximately 30% required occasional external support. While many participants reported limited prior familiarity with audio round-trip latency and related measurement practices, the majority were nevertheless able to complete the corresponding task successfully.

Perceived usability, assessed using the SUS metric, yielded a mean score of 55.8 (SD = 19.3) for the whole test, which is below the standard benchmark score of 68 and corresponds to a marginal level of usability. The wide range of scores (15.0–82.5)^37^ indicates substantial variability in user experience, suggesting that usability issues may affect some users more strongly than others. Perceived workload, measured using the raw (unweighted) NASA-TLX (0–100), resulted in an overall mean score of 37.3 (SD = 13.5), indicating a moderate workload primarily driven by mental demand and effort rather than physical demand. As shown in Table 5, Mental Demand and Frustration had the highest mean scores, whereas Physical Demand was low, consistent with the interaction-based nature of the tasks. Temporal Demand, Effort, and Performance showed intermediate means with substantial variability, reflecting heterogeneous user experiences.

**Table 5:** NASA-TLX results across participants (mean and standard deviation). Higher scores indicate greater workload. The Performance dimension is inversely scaled

Dimension	Mean	SD
Mental demand	54.36	25.22
Physical demand	14.91	15.63
Temporal demand	30.00	17.89
Performance	41.09	32.43
Effort	38.86	26.39
Frustration	44.50	31.24

### Discussion and limitations

Taken together, this preliminary user evaluation indicates that the platform supports its primary objective of enabling collaborative audio recording for dataset creation. High completion rates for recording-related tasks and latency estimation demonstrate that the core functionality can be used successfully by musicians under realistic conditions and without extensive prior training.

Although participants were not explicitly instructed to define privacy levels or user roles during the content creation exercise, these features were implicitly used throughout the collaborative composition tasks. Post-hoc validation of the data generated during the experiment confirmed the presence of multiple privacy settings across compositions. Similarly, assigned collaborator roles varied beyond the default configuration, ranging from Member to Admin. This indicates that participants naturally adjusted both privacy and role parameters without receiving explicit guidance, reflecting an intuitive use of these access-control mechanisms.

At the same time, the evaluation highlights areas for improvement. Tasks involving metadata annotation and collaboration management were more error-prone, suggesting that these features are less immediately discoverable or require clearer conceptual distinctions within the interface. The concentration of errors during the initial stages of the evaluation further indicates a learning curve effect rather than fundamental usability barriers.

Several limitations must be acknowledged. The study involved a limited number of participants and focused on short-term interaction rather than long-term adoption or sustained collaborative use. Furthermore, no qualitative investigation was conducted (e.g., semi-structured interviews or open-ended feedback sessions), which limits deeper insight into participants’ subjective experiences, motivations, and strategies when using the platform. Nevertheless, the post-study questionnaire included two open-ended questions that allowed participants to freely report difficulties encountered during task completion and to provide general feedback on the platform. Analysis of these responses indicates that several issues stemmed from insufficient task prompting and limited discoverability of certain user interface elements. In particular, participants reported ambiguity in task instructions—especially regarding the requirement to record the same musical material multiple times, which led some users to hesitate and improvise alternative workflows. Multiple respondents also highlighted usability issues affecting manual metadata annotation and collaborative actions, such as cloning projects or inviting contributors. These difficulties were consistently attributed to unintuitive placement of controls (e.g., the clone button located in the top-right corner, see Fig. 2), low visual salience, and a navigation structure that some users found confusing or misleading.

Beyond these interface-related observations, the evaluation did not include a comparative assessment against alternative tools. Moreover, the participant sample consisted predominantly of student musicians with relatively strong technical backgrounds, which may limit the generalisability of the findings to less technically experienced or non-student user populations.

Despite these limitations, the combination of objective task performance metrics with subjective usability and workload measures provides a systematic and transparent account of how musicians interact with the platform in practice, thereby complementing the technical contributions presented in this work.

## Conclusions and future work

This article presents Hi-Audio, an open-source online platform designed for the distributed and asynchronous collection of multitrack musical datasets over the internet. The platform is based on a DAW and incorporates features that distinguish it from similar solutions, such as collaborative workflows, content publishing mechanisms, and the implementation of roles and privacy levels.

The article examines the technical aspects of the platform’s implementation, including data model representation, and outlines the various modules that comprise its architecture, clearly differentiating between client-side and server-side components. It focuses on key technological challenges, such as audio constraints, browser memory limitations during audio decoding or the network speed for the upload/download of audio files. To mitigate some of these issues, the platform employs server-side compression techniques to reduce file sizes and ensure a standardized audio format.

Development examples illustrate the complexity of ensuring compatibility across different web browsers—particularly with regard to the fragmentation associated with the Web Audio API. Additionally, the article addresses the problem of round-trip latency estimation, which is critical for the accurate synchronization of audio tracks during recording sessions. A novel and robust solution is proposed for this issue, based on MLS signals and derived from impulse response characterization techniques commonly used in room acoustics.

To enhance the resulting datasets, the platform supports automatic annotation of collected audio tracks. Two machine learning-based approaches are presented: one utilizes the Essentia library to extract objective audio descriptors such as tempo (BPM) and tonality, while the other is based on the AST model for musical instrument recognition and classification.

In addition to the technical contributions presented in this work, a preliminary user evaluation study provides initial empirical evidence that the platform can be effectively used by musicians to perform collaborative audio recording tasks. The results indicate that core recording functionalities are readily usable in realistic conditions, while also highlighting usability challenges related to metadata annotation and collaboration workflows.

Future work will address the usability issues identified in the evaluation through improved onboarding mechanisms and interface refinements. Moreover, larger-scale and longitudinal user studies involving more diverse participant profiles will be conducted to further assess long-term adoption, collaborative practices, and dataset quality in real-world scenarios.

Future development of the platform will focus on enhancing functionality and user experience through three main directions. First, content review and moderation mechanisms will be introduced to allow composition owners to manage visibility and control contributions in open collaborative settings. Second, a search engine will be developed to enable efficient content retrieval through free-text or keyword-based queries based on the metadata associated with stored audio content. Third, the platform will integrate new tools and methods for music content analysis, with particular emphasis on automatic and semi-automatic content annotations that are temporally aligned with the audio tracks. Such annotations may encompass rhythmic patterns, melodic and harmonic structures, chord progressions, offensive content, or other salient auditory events. Together, these developments are expected to enhance collaborative workflows for data collection and analysis, while improving the accuracy and generalizability of music information retrieval tasks, thereby strengthening the robustness and applicability of the proposed framework.

In parallel with these technical enhancements, future work will also focus on leveraging the curated database for targeted research applications. The availability of isolated tracks for each composition—recorded independently and free from bleeding by other instruments, creates valuable opportunities for experiments in audio source separation, automatic mixing and demixing, generative audio modeling, cover version detection, and music style transfer. This structured and interference-free dataset positions the platform as a useful resource for advancing state-of-the-art techniques in music signal processing and machine learning.

## Availability and requirements

 

- Project name: Hi-Audio Online Platform
- Project home page: https://hiaudio.fr/
- Operating system(s): Web Application, OS independent
- Programming language: JavaScript, HTML, CSS and Python
- The following browser versions are recommended: Firefox ($\ge 79.0$), Chromium-based ($\ge 78.0$), Safari ($\ge 14.1$)
- Source Code License: MIT
- Music License: Creative Commons Attribution 4.0 International.

## Data Availability

All source code developed is open and available at https://hiaudio.fr/static/github.html. Music data created or generated during the study is also accessible at https://hiaudio.fr. The following datasets warrant particular attention: $\bullet$ The dataset generated during the current study for the HAPPY BIRTHDAY collection is available at: https://hiaudio.fr/index.html?collectionid=n5hoJCqxToAMAtKwFgBide. $\bullet$ The datasets analyzed during the current study for accuracy estimation of the AST model are available in the DSD100 and MoisesDB repositories, at https://sigsep.github.io/datasets/dsd100.html and https://music.ai/research/, respectively. $\bullet$ All the results and analysis from the data generated by the User Evaluation are available at: https://github.com/idsinge/hiaudio_user_evaluation_results.

## Footnotes

1 https://www.ismir.net/resources/datasets/

2 Western music refers here to occidental musical traditions originating primarily from Europe and North America, typically characterized by tonal harmony, staff notation, and standardized tuning systems

3 https://www.go-fair.org/fair-principles/

4 https://hi-audio.imt.fr/agenda/29th-april-music-workshop/

5 https://audiomovers.com/listento/

6 https://jamkazam.com/

7 https://www.cockos.com/ninjam/

8 https://creativecommons.org/licenses/by/4.0/

9 https://www.avid.com/solutions/music-creation

10 https://www.musesessions.co/

11 https://mixedinkey.com/satellite/

12 https://www.kompoz.com/

13 https://opendaw.org/

14 https://github.com/bitwig/dawproject

15 https://moises.ai/

16 https://gdpr.eu/

17 Root Mean Square

18 https://hiaudio.fr/static/support.html

19 https://hiaudio.fr/index.html?collectionid=n5hoJCqxToAMAtKwFgBide

20 A polyfill is code that implements a feature or enables the use of an API regardless of whether it is supported by a web browser.

21 https://getbootstrap.com/docs/4.6/getting-started/introduction/

22 https://github.com/naomiaro/waveform-playlist

23 https://wavesurfer.xyz/

24 https://github.com/gilpanal/waveform-playlist

25 https://github.com/idsinge/hiaudio_import

26 https://github.com/bmartel/audio-file-decoder

27 https://github.com/soundcut/decode-audio-data-fast

28 https://github.com/eshaz/wasm-audio-decoders

29 The prototype is provided in a separate publicly accessible repository to facilitate detailed study and analysis https://github.com/gilpanal/weblatencytest

30 https://flask.palletsprojects.com/en/2.3.x/

31 https://pytorch.org/

32 https://github.com/gilpanal/instrument_recognition_AST

33 https://acoustid.org/

34 https://github.com/jiaaro/pydub/

35 https://caniuse.com/?search=audio%20format

36 https://lascene.rezel.net/

37 https://github.com/idsinge/hiaudio_user_evaluation_results
